# Phylogenetic placement of *Ceratophyllum submersum* based on a complete plastome sequence derived from nanopore long read sequencing data

**DOI:** 10.1186/s13104-023-06459-z

**Published:** 2023-08-25

**Authors:** Samuel Nestor Meckoni, Benneth Nass, Boas Pucker

**Affiliations:** 1 Plant Biotechnology and Bioinformatics, Institute of Plant Biology, TU Braunschweig, 38106 Braunschweig, Germany; 2 Faculty of Environmental Sciences, Czech University of Life Sciences Prague, Kamýcká 129, Praha 6 - Suchdol, CZ-165 21 Prague, Czech Republic; 3 Braunschweig Integrated Centre of Systems Biology (BRICS), TU Braunschweig, 38106 Braunschweig, Germany

**Keywords:** ONT, Angiosperms, Ceratophyllaceae, Ceratophyllales plastome, Plastomics, Chloroplast-genome phylogenomics, Phylogenetic pipeline, Supermatrix tree

## Abstract

**Objective:**

Eutrophication poses a mounting concern in today’s world. *Ceratophyllum submersum* L. is one of many plants capable of living in eutrophic conditions, therefore it could play a critical role in addressing the problem of eutrophication. This study aimed to take a first genomic look at *C. submersum*.

**Results:**

Sequencing of gDNA from *C. submersum* yielded enough reads to assemble a plastome. Subsequent annotation and phylogenetic analysis validated existing information regarding angiosperm relationships and the positioning of Ceratophylalles in a wider phylogenetic context.

**Supplementary Information:**

The online version contains supplementary material available at 10.1186/s13104-023-06459-z.

## Introduction

**Fig. 1:**
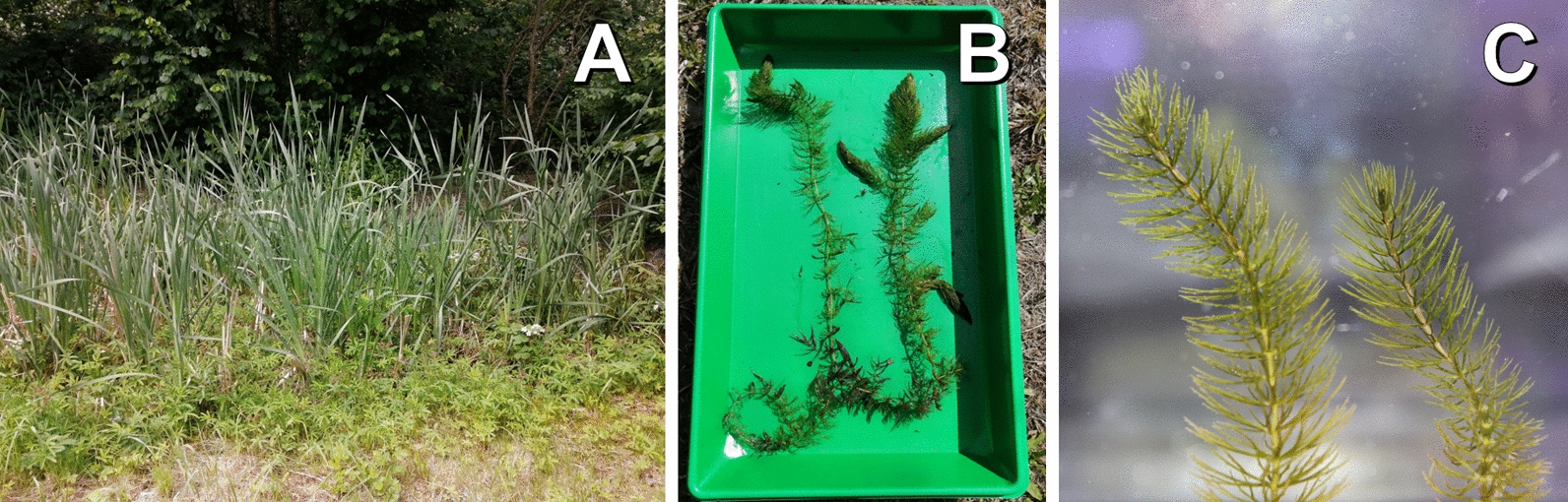
***Ceratophyllum submersum*** and its habitat. **A**: The pond (Braunschweig, 52.28062 N / 10.54896 E) where *C. submersum* was collected, **B**: Whole *C. submersum* plant, **C**: Close up view of *C. submersum*

*Ceratophyllum submersum* L., commonly known as soft hornwort, is a subaquatic plant whose genus is the only extant member in the order of Ceratophyllales, placed as a sister clade to the eudicots [1, 2]. It is native to Europe, Africa and Asia and grows in stagnant freshwater bodies [3]. Morphological features include long, branching stems that can reach up to several metres in length and leaves that are forked into narrow, filament-like segments that grow in multiple whorls around the stem (Fig. 1) [4]. The plant is often green, but can vary in colour from brown to red depending on environmental conditions. Anthocyanins contribute to the colouration of many plant species, and metabolic analyses have detected several derivatives in *C. submersum* [5–7]. It thrives in eutrophic conditions, characterised by low light intensity and high nutrient levels [8, 9]. Eutrophication of aquatic environments is indicated by the accumulation of nutrients albeit other parameters and multiple classification systems exist [10]. Due to anthropogenic effects, the occurrences of eutrophic environments are rising which poses a problem e.g. for greenhouse gas emissions [11]. In aquatic systems, eutrophication induces harmful algal blooms (HABs) which are responsible for environmental hazards like the Oder ecological disaster in 2022 [12]. Due to its capabilities, *C. submersum* competes with other phototrophic organisms capable of living in eutrophic conditions. This suggests that it may inhibit the formation of HABs despite its vulnerability to them [7].

While a low coverage skimming report has been performed previously [13], the results appear to be missing in the established sequence databases. Here, we utilised nanopore long-read sequencing to obtain the plastome sequence of *C. submersum* and provide a thorough annotation of its genetic content. This analysis allowed us to place the species in a phylogenetic context which informs future studies.

## Results and discussion

A total of 0.48 Gbp of sequencing data was generated from 94,100 long reads. Out of this, 1,544 reads representing the *C. submersum* plastome were extracted (Additional file 1, PRJEB62706), accounting for 3.3% of the data. Due to the limitations of the Flye assembler used by ptGAUL, only 752 reads (covering about 1.68% of the total data) were utilised for the assembly, as it can only accommodate up to 50x coverage of the estimated assembly size. Considering these factors, only 0.27 Gbp of sequencing data are required to assemble an approximately 160 kbp sized plastome, provided that the ratio of plastid DNA to nuclear DNA does not exceed 3%. We calculated that about 0.8 Gbp of total genomic sequencing data would be adequate for cases where the plastid DNA content is even lower (1%). High-quality assemblies can be achieved with coverage levels lower than 50, so a smaller amount of data may still be sufficient. We inferred a sequencing goal of 1 Gbp to enable the assembly of a plastome sequence without prior plastid purification. If fresh leaf material is chosen, a higher plastid DNA portion should be achievable, potentially reducing the amount of data needed for successful assembly.

The plastome assembly of *C. submersum* resulted in a 155,767 bp long sequence with a GC content of 38.26%. In total, 75 unique protein coding genes were annotated (Additional file 2). The *C. submersum* plastome sequence is 485 bp shorter than the reference plastome sequence of *Ceratophyllum demersum* L. that has a length of 156,252 bp [14]. While the GC content is similar (*C. demersum*: 38.22, *C. submersum*: 38.26) the amount of unique annotated genes in *C. submersum* is smaller than in *C. demersum* (75 against 79). After the annotation some predicted coding sequences were flawed (e.g. containing multiple stop codons). Manual evaluation revealed four homopolymeric regions in which a frameshift would correct the annotation. Since homopolymers are a frequent error type in ONT reads, manual correction in those regions is appropriate [15]. The comparison of those regions to all plastome reads suggested a correction of two of these regions, namely the adenine at position 3,094 and the thymines at position 3,143, at position 86,261, and at position 86,262 were inserted.

**Fig. 2:**
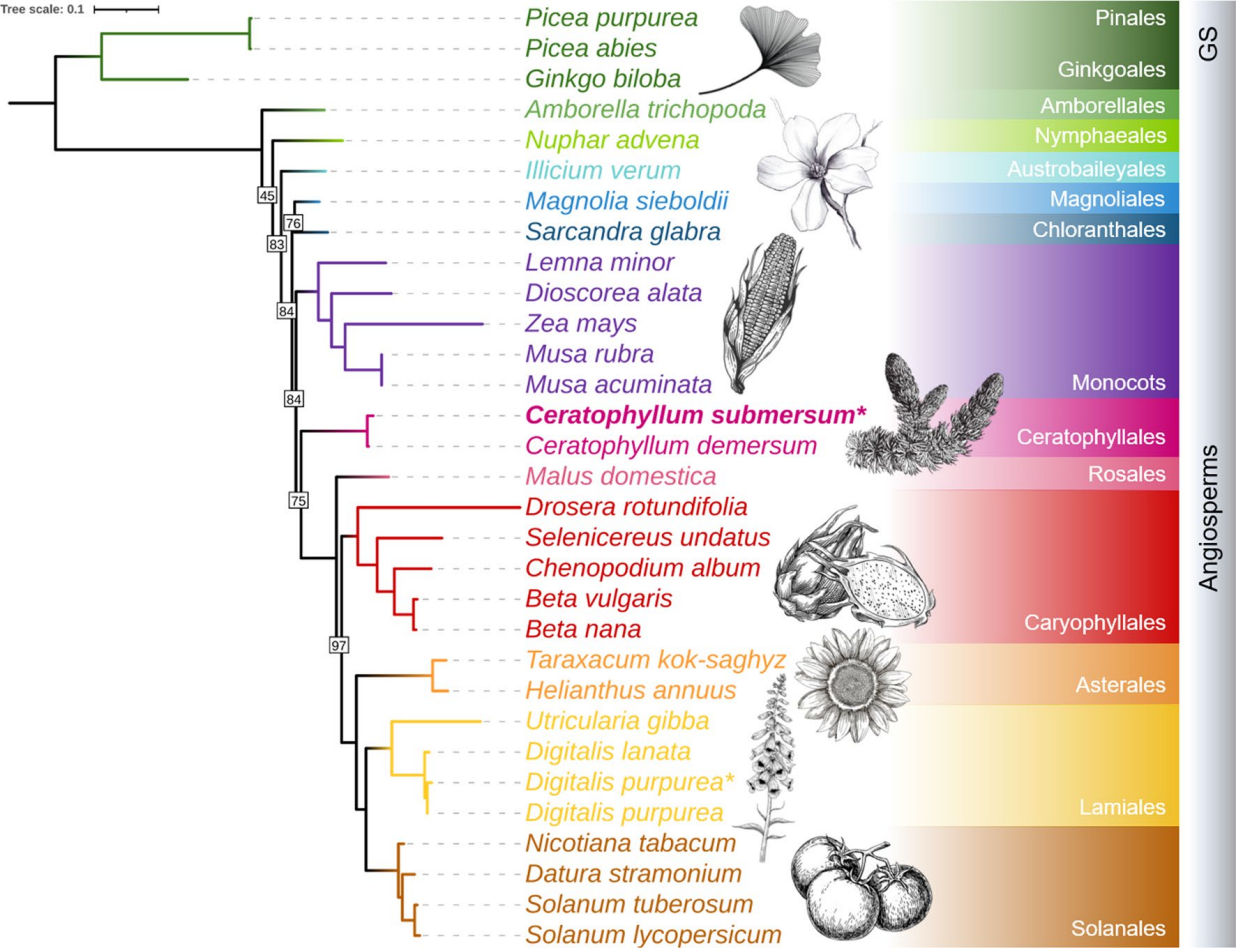
Phylogenetic tree of selected spermatophytes based on plastome protein sequences. The asterisks (*) indicate plastome assemblies generated in this study. Within the angiosperms, the clades are differently coloured. All nodes received full bootstrapping support (100%), except those displaying the actual value. Please see the material and methods section for further description of the tree calculation. Visualisation was done in iTOL 6.7.6 [16]. The full tree including the outgroup *Chlamydomonas reinhardtii* can be found in the additional files (Additional file 4). GS = gymnosperms. The sketches are licensed by adobe and depositphotos (Standard License: https://wwwimages2.adobe.com/content/dam/cc/en/legal/servicetou/Stock-Additional-Terms_en_US_20221205.pdf, https://depositphotos.com/license.html)

The sequencing took place on a flow cell that was previously used to analyse gDNA from *Digitalis purpurea* L. Therefore, a phylogenetic tree was calculated to validate clean and distinguishable sequencing data, incorporating our plastome assemblies for both species (*D. purpurea* data: PRJEB62706) (Fig. 2).

Multiple plastome reference sequences were chosen to represent the angiosperm clade as well as *Chlamydomonas reinhardtii* as outgroup (for full list and references see Additional file 3). The phylogenetic tree (Fig. 2, for full tree see Additional file 4) classifies *C. submersum* close to its reference *C. demersum* and our *D. purpurea* plastome assembly close to the RefSeq *D. purpurea* plastome sequence generated by Zhao et al. [17]. The angiosperm clade is represented in accordance with the current APG IV classification except for the exact separation/placement of the Magnoliids and the Chloranthales, which is still controversial [2]. This underlines the significance of plastome sequences for modern plant phylogenetics [18].

The plastome assembly and phylogenetic analysis presented in this study provides first steps towards genetic and genomic characterization of *Ceratophyllum submersum*. Further research is needed to determine its nuclear genome and to explore potential applications of this plant, such as its use as a valuable resource or as an agent to mitigate environmental hazards.

## Materials and methods

### Plant material, gDNA extraction and sequencing

*Ceratophyllum submersum* was collected from a small pond in Braunschweig (52.28062 N / 10.54896 E) and kept in cultivation at the Institute of Plant Biology at TU Braunschweig. The artificial pond needs occasional water replenishment. Foliage from surrounding shrubs and dead aquatic plants lead to a high eutrophy (see Fig. 1A). *C. submersum* shoot tips (Fig. 1C) were harvested for gDNA extraction conducted with a CTAB method [19–21]. Short DNA fragments were depleted with the Short Read Eliminator kit (Pacific Biosciences). Library preparation for ONT sequencing was started with 1 µg of DNA that was first repaired with the NEBNext^®^ Companion Module and then processed according to the SQK-LSK109 protocol (Oxford Nanopore Technologies). For ONT sequencing, a R9.4.1 flow cell was used with a MinION. ONT sequencing is one of the leading sequencing technologies in plant genomics [22], and was applied in this project to generate a complete plastome sequence based on long reads. Prior to *C. submersum* sequencing, the flow cell was already utilised for *D. purpurea* gDNA sequencing. Processing of raw data was performed with guppy v6.4.6 + ae70e8f (https://community.nanoporetech.com) which internally called minimap2 v2.24-r1122 [23] with default parameters on a graphical processor unit (GPU) in the de.NBI cloud to generate FASTQ files. Guppy was run with the default configuration of dna_r9.4.1_450bps_hac.

### Plastome assembly and annotation

The FASTQ files were subjected to a plastome assembly with ptGAUL using standard parameters [23–26]. As reference the *C. demersum* plastome from the NCBI RefSeq database (release 216) was used [14, 27]. Since the ptGAUL results consist of two assemblies (one per path) the OGDRAW plastome maps were compared between the reference and the assemblies to decide which one is closer to the reference. The *C. submersum* assembly needed to be reverse complemented and the sequence start was adjusted to the *C. demersum* sequence. GC contents of the assembly were calculated using the contig_stats.py script [28]. For annotation, GeSeq was used (see Additional file 5 for parameters) [29–38]. Protein coding genes were extracted from the resulting GBSON.json file using our own script JSON_2_peptide_fasta_v1.0.py [39]. Frameshifts in the coding sequences of incorrectly translated peptide sequences were identified in two steps. First, all open reading frames (ORFs) were annotated by EMBOSS sixpack. Then, NCBI blastx results were mapped against the ORFs to specify the location of the possible frameshifts [40, 41]. Further evaluation of these frameshifts was conducted with the help of a read mapping. All plastome reads (FASTQ) were mapped against the plastome sequence (FASTA) with minimap2 (v2.24-r1122, parameters: -ax map-ont --secondary = no -t 27) to generate a SAM file [23]. From that, a BAM file and its corresponding index file was generated with samtools 1.10 [42]. The mapping was then analysed in the Integrative Genomics Viewer (IGV) 2.16.1 [43]. The assembly underwent correction only if supported by more than a quarter and a minimum of ten reads.

Data preparation prior to submission was performed by extracting all the read IDs from the ‘new_filter_gt3000.fa’, generated by ptGAUL, which contains all the identified plastome reads. The plastome reads were extracted from the FAST5 and FASTQ raw read datasets via the ‘fast5_subset’ command from the ont_fast5_api tool and our custom script FASTQ_extractor_from_FAST5_mapping_file.py (both using default parameters) [39, 44].

### Phylogenetic analysis

Reference data retrieval and phylogenetic supermatrix tree construction was performed by our newly developed Python pipeline PAPAplastomes (Pipeline for the Automatic Phylogenetic Analysis of plastomes). It integrates established external tools for complex steps (Additional file 6) [45]. Reference species, closely related species of interest, and outgroup species can be specified via a config file. First, NCBI RefSeq plastome data is downloaded and the reference plastome peptide sequences are extracted from this collection. These peptide sequences are further combined with the peptide sequences derived from the assemblies representing plastomes of interest. The pre-OrthoFinder trimming step removes potential paralogs with the exact same sequence, sequences which contain asterisks, and sequences that are shorter than 10 amino acids (this threshold value is adjustable). Next, OrthoFinder v2.5.4 [46–49] is applied. Post-Orthofinder processing includes four steps. Removal of outlier sequences (first step), deletion of orthogroups missing the species of interest or their references (second step), removal of orthogroups harbouring fewer species than the pre-defined outgroup species (third step), and paralog cleaning (fourth step). These steps are explained in more detail below. First step: Since the OrthoFinder results include phylogenetic trees of each orthogroup, outlier identification is conducted with the help of the Python module Dendropy v4.5.2 [50]. Per orthogroup the edge length for each taxon is accessed except for outgroup species. Based on these lengths, outliers are identified by the 1.5*IQR method (inter quartile range) i.e. sequences with a distance larger than 1.5 times the variation are excluded. Second step: The species of an orthogroup are listed and if pre-defined species are all either present or absent, the orthogroup will be kept. Otherwise, the orthogroup will be discarded. This is suggested for closely related species i.e. from the same genus and can be specified by the user in the config file. Third step: Orthogroups consisting solely of the outgroup species are exempt from this criterion. Fourth step: Among remaining paralogs within one species, only the longest sequence is kept.

The cleaned orthogroups are then aligned with MAFFT v7.453 (--maxiterate 1000 --localpair) [51, 52] and alignments are concatenated. Phylogenetic supermatrix tree calculation is performed by IQ-TREE (multicore version 1.6.12 for Linux 64-bit built Aug 15 2019; with the ‘-nt AUTO -bb 1000’ options, seed: 291,752) based on the concatenated alignment of all orthogroups [53–55].

### Limitations

Before sequencing *C. submersum*, the flow cell had already been utilised for sequencing gDNA of *D. purpurea*. Insufficient DNA availability hindered the complete sequencing and assembly of *C. submersum*’s nuclear genome. Future optimisations in DNA extraction methods dedicated to small aquatic plants could overcome this limitation.

### Supplementary Information

**Additional file 1.** FASTQ stats of the Ceratophyllum submersum plastome reads. The stats were calculated with the help of the script FASTQ_stats3.py (https://github.com/bpucker/GenomeAssembly).

**Additional file 2. **Ceratophyllum submersum plastome map derived from OGDRAW.

** Additional file 3.** List of references of the reference plastomes. The data was extracted by PAPAplastomes and originates from the RefSeq database.

**Additional file 4. **Full phylogenetic tree with outgroup species Chlamydomonas reinhardtii.

**Additional file 5.** Exact parameters for the GeSeq run.

**Additional file 6.** PAPAplastome workflow chart.

## Data Availability

The datasets generated and analysed during the current study are available under PRJEB62706. Scripts developed for the data analysis are available from Codeberg (https://codeberg.org/snmeckoni/scripts, https://codeberg.org/snmeckoni/PAPAplastomes) and GitHub (https://github.com/bpucker/GenomeAssembly).

## Abbreviations

CDS: Coding sequence
gDNA: Genomic DNA
GPU: Graphical processing unit
GS: Gymnosperm
HAB: Harmful algal bloom
RefSeq: NCBI Reference Sequence Database
IQR: Interquartile range
ONT: Oxford Nanopore Technologies
