# Supplementary figures and images for: Phylogenetic placement of Ceratophyllum submersum based on a complete plastome sequence derived from nanopore long read sequencing data
Source: BMC Res Notes. 2023 Aug 25;16:187. doi: 10.1186/s13104-023-06459-z (PMC10464454; doi:10.1186/s13104-023-06459-z)

**Additional file 2: *Ceratophyllum submersum* plastome map derived from OGDRAW.**

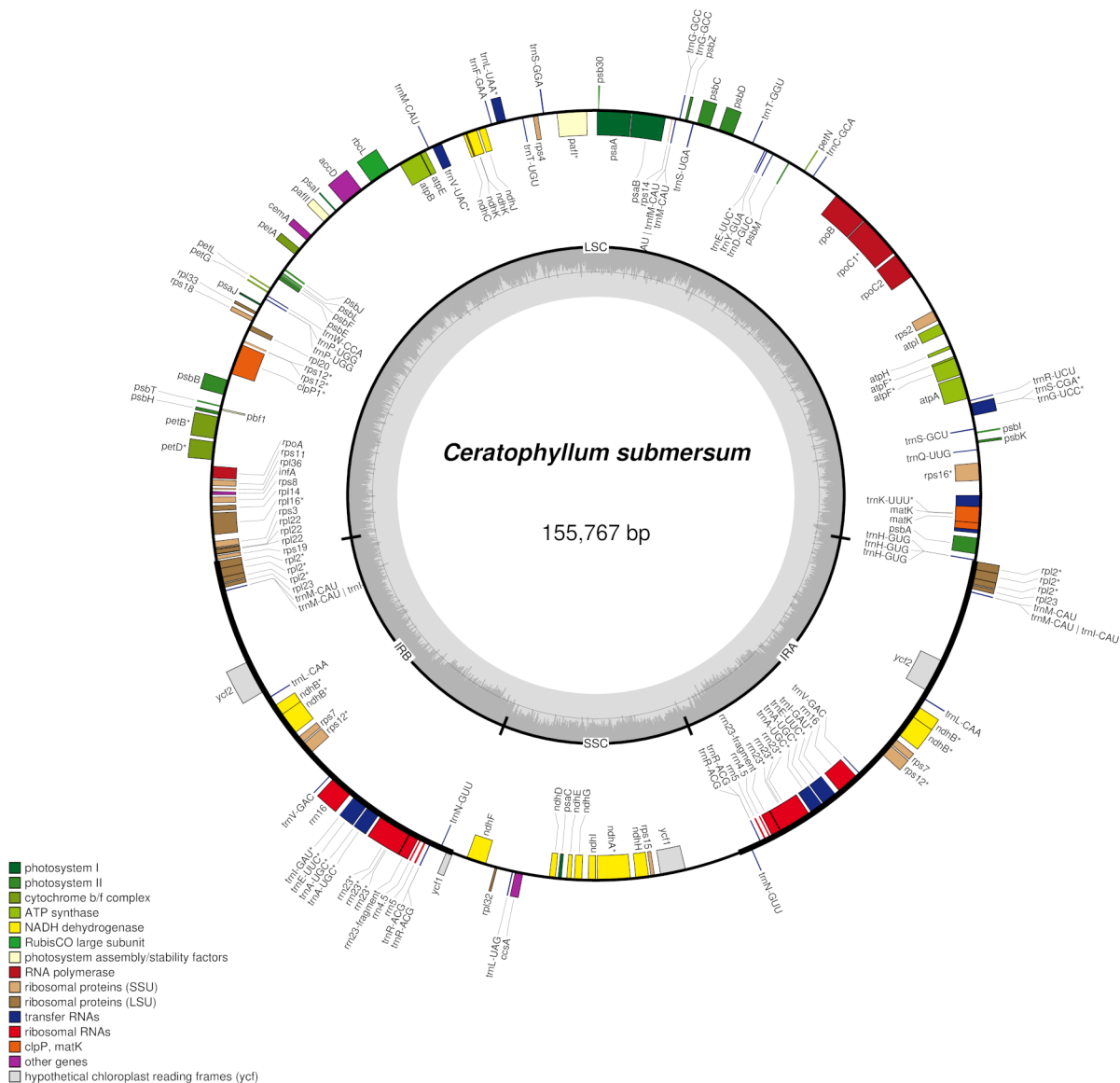

Supplement: Supplementary file 2 — Additional file 2. Ceratophyllum submersum plastome map derived from OGDRAW. [file 13104_2023_6459_MOESM2_ESM.pdf]

# Additional file 4: Full phylogenetic tree with outgroup species *Chlamydomonas reinhardtii*.

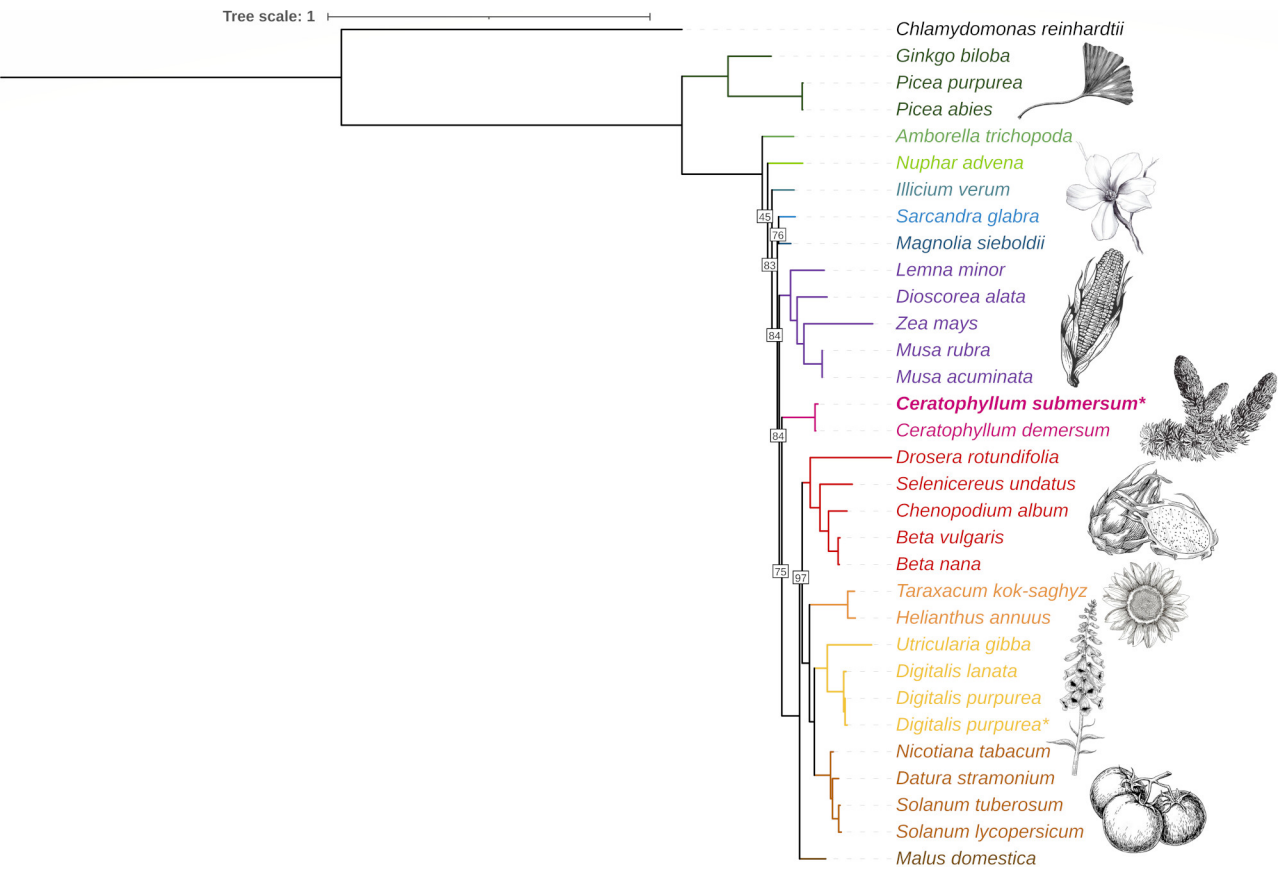

Supplement: Supplementary file 4 — Additional file 4. Full phylogenetic tree with outgroup species Chlamydomonas reinhardtii. [file 13104_2023_6459_MOESM4_ESM.pdf]

# Additional file 6: PAPAplastome workflow chart.

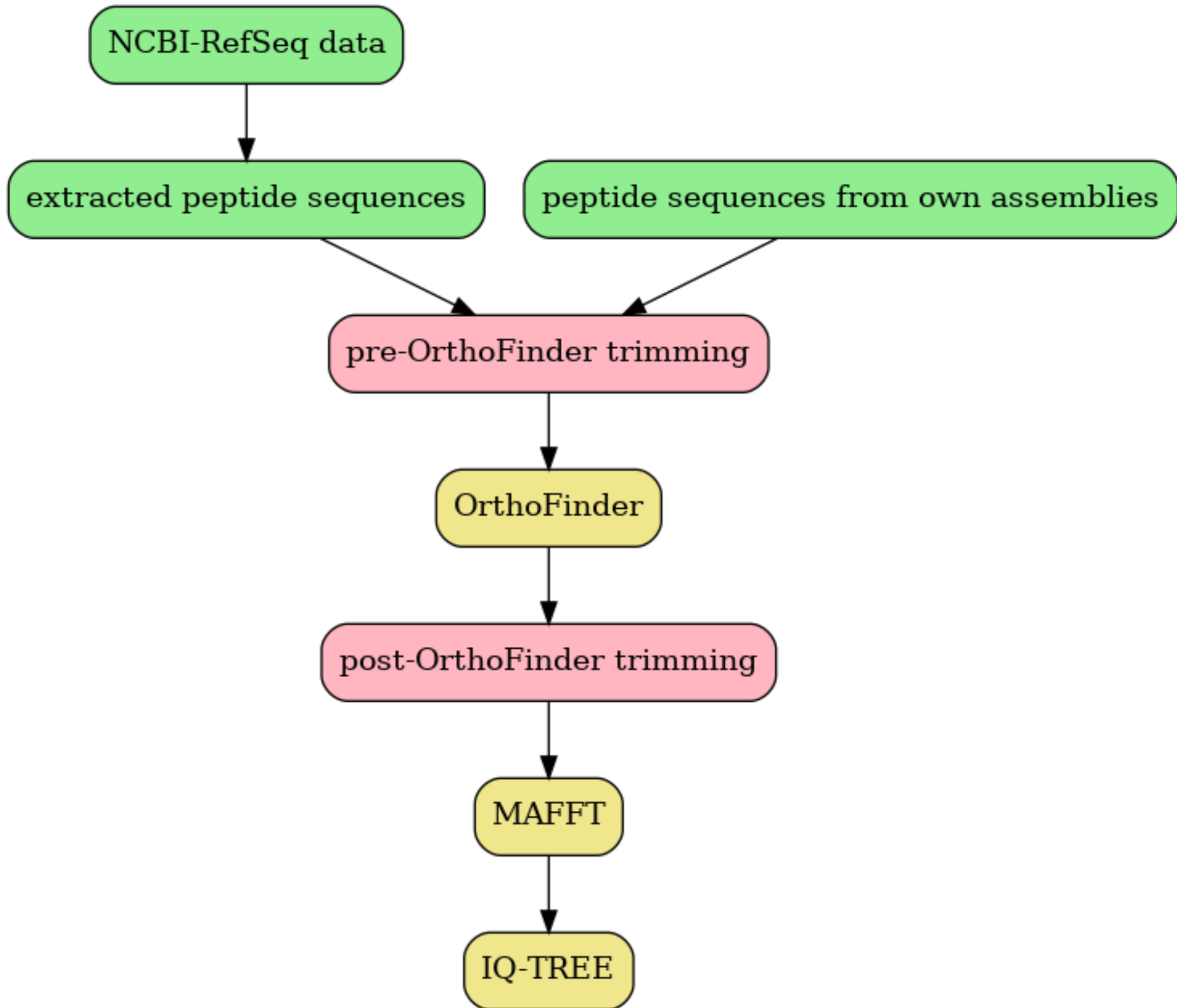

Supplement: Supplementary file 6 — Additional file 6. PAPAplastome workflow chart. [file 13104_2023_6459_MOESM6_ESM.pdf]
